# Prevalence of *Aggregatibacter actinomycetemcomitans* and Periodontal Findings among 14 to 15-Year Old Danish Adolescents: A Descriptive Cross-Sectional Study

**DOI:** 10.3390/pathogens9121054

**Published:** 2020-12-16

**Authors:** Anne Birkeholm Jensen, Flemming Isidor, Marianne Lund, Michael Væth, Anders Johansson, Niels Nørskov Lauritsen, Dorte Haubek

**Affiliations:** 1 Section for Pediatric Dentistry, Department of Dentistry and Oral Health, Health, Aarhus University, 8000 Aarhus, Denmark; abj@dent.au.dk; 2 The Municipality of Aarhus, 8000 Aarhus, Denmark; 3 Section for Prosthetic Dentistry, Department of Dentistry and Oral Health, Health, Aarhus University, 8000 Aarhus, Denmark; 4 Department of Clinical Microbiology, Aarhus University Hospital, Skejby, 8210 Aarhus, Denmark; marialun@rm.dk; 5 Department of Public Health, Aarhus University, 8000 Aarhus, Denmark; vaeth@ph.au.dk; 6 Divison of Molecular Periodontology, Department of Odontology, Faculty of Medicine and Odontology, Umea University, 901 87 Umea, Sweden; anders.p.johansson@umu.se; 7 Department of Clinical Microbiology, Odense University Hospital, 5000 Odense, Denmark; nielnoer@rm.dk

**Keywords:** periodontitis, JP2 genotype, periodontal outcome, *A. actinomycetemcomitans*, saliva sample, subgingival plaque sample, leukotoxin

## Abstract

*Aggregatibacter actinomycetemcomitans* (*Aa*) is a keystone pathogen associated with periodontitis in adolescents. The knowledge on the prevalence of *Aa* and periodontitis among adolescents in Northern Europe is sparse. A total of 525 14- to 15-year-old adolescents from the municipality of Aarhus, Denmark, underwent a full-mouth clinical examination. Plaque score (PS), bleeding on probing (BOP), probing pocket depth (PPD), and clinical attachment loss (CAL) were recorded. Subgingival plaque samples (SPS) and stimulated saliva samples (SSS) were collected and analyzed for the presence of JP2 and non-JP2 genotypes of *Aa* using real-time PCR. A total of 70 (13.3%) individuals were positive for *Aa*, with 17 found in SPS, 19 in SSS, and 35 in both. The highly leukotoxic JP2 genotype of *Aa* was not detected. The individuals positive for *Aa* in both SPS and SSS had poorer periodontal outcomes (PPD and CAL) than individuals without *Aa* and individuals carrying *Aa* in either SPS or SSS only. In conclusion, 13% of 14- to 15-year-old Danish adolescents were positive for *Aa*, and the presence of *Aa* in both SPS and SSS was associated with poorer periodontal outcomes.

## 1. Introduction

The facultative anaerobic Gram-negative rod *Aggregatibacter actinomycetemcomitans* is recognized as a keystone pathogenic bacterium involved in the pathogenesis of periodontitis, especially in young individuals [1], and the highly leukotoxic JP2 genotype of *A. actinomycetemcomitans* has been reported to significantly increase the risk of developing clinical attachment loss early in life [2,3,4]. The JP2 genotype of *A. actinomycetemcomitans* frequently occurs in young individuals living in Northwest Africa [5], and the prevalence of different genotypes of *A. actinomycetemcomitans* is reported to depend on geography [6,7]. A considerable amount of research has been done on the prevalence of *A. actinomycetemcomitans* and periodontal disease in African, American, Asian, and European countries [3,8,9,10,11,12,13,14,15,16,17,18,19,20,21], but studies undertaken in Northern European countries on this topic are sparse [22,23,24]. Demographics in many European countries are changing due to human migration. Therefore, the occurrence of *A. actinomycetemcomitans* and the prevalence of periodontal disease are interesting topics to study. The association between the carrier status of *A. actinomycetemcomitans* and development of periodontal disease could be different in Northern European populations compared to, e.g., African populations due to general health status, genetics, accessibility to dental care, and oral hygiene habits. Surprisingly, a study of Swedish periodontitis patients reported 30% *A. actinomycetemcomitans* carriers among diseased individuals [23]. Furthermore, the detection of the JP2 genotype of *A. actinomycetemcomitans* in Caucasian individuals was reported on [23]. Interestingly, in addition to the JP2 genotype, other relatively high-leukotoxic genotypes with various leukotoxin promoter modifications were detected in this population [25]. Hence, additional population-based research on the prevalence of *A. actinomycetemcomitans*, and especially the highly leukotoxic genotypes of *A. actinomycetemcomitans*, is needed in Northern Europe.

The aim of this study was to describe the presence of the periodontal keystone pathogen *A. actinomycetemcomitans* in a Danish adolescent population, measured in subgingival plaque and in stimulated saliva. Groups defined by carrier status of *A. actinomycetemcomitans* were generated, described, and analyzed according to periodontal outcomes.

## 2. Results

### 2.1. Study Population

With written consent, a total of 525 individuals agreed to participate in the present study. The distribution between genders was even, with participation of 260 boys and 261 girls (one individual did not answer the questionnaire, two did not specify their gender, and one individual provided one biological sample only). Three individuals reported having diabetes. Five hundred and eight (96.7%) defined themselves as healthy, 64 (12.2%) had a daily consumption of various types of medicine, and 29 (5.5%) of the participants had an intake of antibiotics within the last three months. Twenty (3.8%) defined themselves as smokers.

### 2.2. Carrier Status of A. actinomycetemcomitans

**Figure 1:**
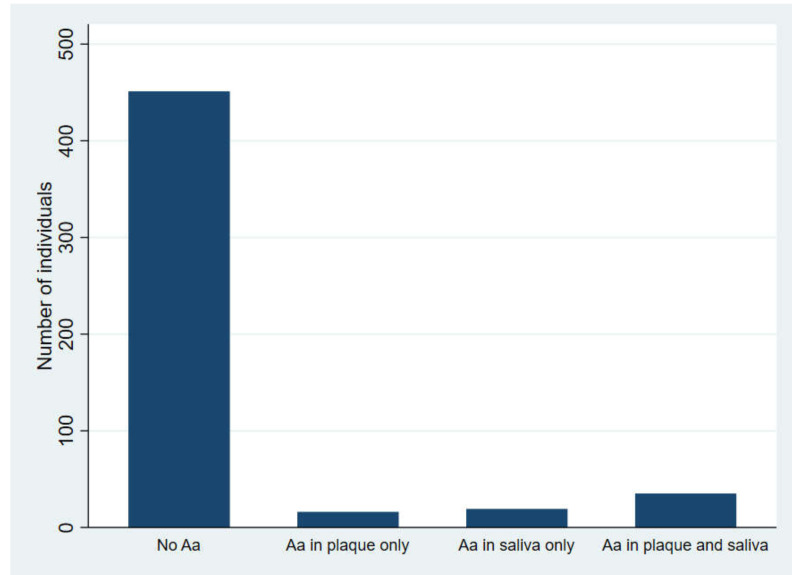
Distribution of participants according to carrier status of *A. actinomycetemcomitans* (*Aa*).

Seventy (13.3%) individuals were positive for *A. actinomycetemcomitans* in either subgingival plaque, in stimulated saliva, or in both. Fifty-one (9.7%) and 54 (10.2%) were positive in subgingival plaque and stimulated saliva, respectively. Of these, 35 were positive in both samples. Seventeen were positive in subgingival plaque samples only, and 19 in stimulated saliva samples only (Figure 1).

The JP2 genotype of *A. actinomycetemcomitans* was not detected in the present study population. No difference was found between genders according to carrier status of *A. actinomycetemcomitans* (*p* > 0.05).

**Figure 2:**
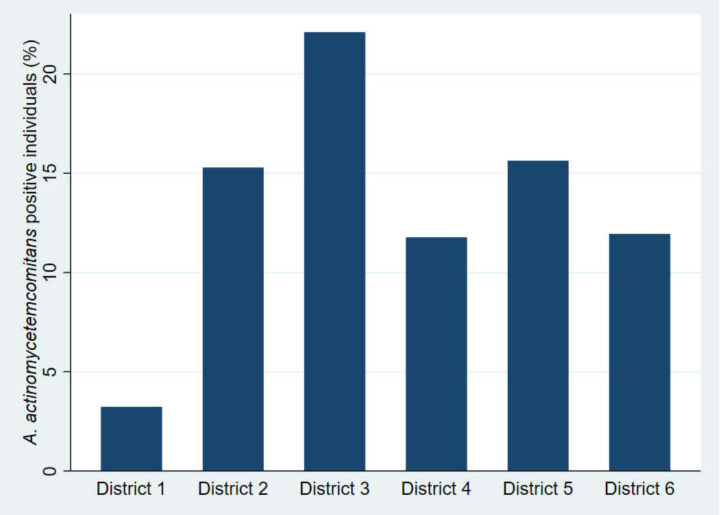
The number of individuals positive for *A. actinomycetemcomitans* (*Aa*) according to the districts of the municipality of Aarhus.

The number of individuals positive for *A. actinomycetemcomitans* according to the districts in the municipality of Aarhus is shown in Figure 2. The highest amount of *A. actinomycetemcomitans*-positive individuals (*n* = 21) was found in district 3, and the lowest number of *A. actinomycetemcomitans*-positive individuals (*n* = 3) was found in district 1.

Table 1 lists the demography of the districts according to ethnic background (data obtained from citizen data from the municipality of Aarhus) [26]. District 1 had the highest number of individuals with a Danish ethnic background (93.9%), and district 3 had the lowest number of individuals with a Danish ethnic background (59.4%). The percentage of individuals positive for *A. actinomycetemcomitans* in each district, adjusted according to the size of the districts in relation to the size of the municipality, showed the same pattern. The relative percentage of *A. actinomycetemcomitans*-positive individuals was 4.3%, 15.7%, 30.0%, 17.1%, 21.4%, and 11.4% from districts 1 to 6, respectively. The difference in prevalence of *A. actinomycetemcomitans* between the districts was statistically significant (*p* < 0.05).

### 2.3. Periodontal Outcomes of the Participants According to A. actinomycetemcomitans-Carrier Status

Data on plaque scores (PS), bleeding on probing (BOP), periodontal probing depth (PPD), and interdental clinical attachment loss (CAL) scores according to the carrier status of *A. actinomycetemcomitans* are shown in Table 2 and Table 3. In the group of individuals positive for *A. actinomycetemcomitans* in subgingival plaque and/or stimulated saliva, there was statistically significantly more individuals having PPD ≥ 4 mm and interdental CAL ≥ 2 mm than in the group of individuals negative for *A. actinomycetemcomitans* (Table 2).

The group of individuals without *A. actinomycetemcomitans* consisted of a statistically significantly lower percentage of individuals having PPD ≥ 4 mm and interdental CAL ≥ 2 mm than the other groups presented in Table 3. The group of individuals positive for *A. actinomycetemcomitans* in both subgingival plaque and stimulated saliva consisted of a statistically significantly higher percentage of individuals with PPD ≥ 4 mm and CAL ≥ 2 mm (Table 3).

The group of individuals positive for *A. actinomycetemcomitans* in stimulated saliva only showed the lowest percentage of individuals with PS > 20%, BOP > 10%, and interdental CAL ≥ 2 mm (Table 3). However, the differences were not statistically significant (Table 3).

## 3. Discussion

*A. actinomycetemcomitans* may be a causative agent in periodontal disease and in non-oral diseases, such as infectious endocarditis, various types of abscesses, bacteremia, osteomyelitis, skin infections, and urinary tract infections [27]. In the present study, we report a prevalence of 13% *A. actinomycetemcomitans*-carriers in a 14- to 15-year-old adolescent population in Denmark, but the highly leukotoxic JP2 genotype strain of *A. actinomycetemcomitans* was not detected. These results suggest an association between being a carrier of *A. actinomycetemcomitans* in both subgingival plaque and stimulated saliva and having poorer periodontal health.

The prevalence of *A. actinomycetemcomitans* is high in Asian and African populations, but is considerably lower in Europe [2,3,8,9,10,11,12,13,14,15,16,17,18,19,20,21,28,29]. The results of the present study, with a prevalence of 13% *A. actinomycetemcomitans*-carriers, defined as individuals positive for *A. actinomycetemcomitans* in subgingival plaque, stimulated saliva, or in both, are comparable to previous findings in European populations [9,11,13,21,24,29]. Alaluusua and co-workers reported 13% of their population of Finnish individuals to be positive for *A. actinomycetemcomitans* [29], and the prevalences found in Switzerland and Italy are 20% and 16%, respectively [9,14]. Belstrøm and co-workers reported a very low occurrence of *A. actinomycetemcomitans* (under 3%) among Danish adult individuals in a case–control study, including both healthy and diseased individuals [22]. *A. actinomycetemcomitans* is a stabile colonizer [30,31,32,33] found more often in young individuals than in adults [34], and the association with periodontitis is primarily reported in the young age groups [2,3,4]. These facts might explain the difference between the results of the present study and the study conducted by Belstrøm and co-workers [22].

District 3 accounted for the highest prevalence of *A. actinomycetemcomitans* (Figure 2), and district 3 represented the part of the municipality with the highest proportion of individuals with a non-Danish ethnic background (Table 1). Other researchers have reported the same tendency [4,6]. However, the proportion of individuals with a non-Danish ethnic background is higher in the municipality of Aarhus compared to the general demographic picture in Denmark [35], and the actual prevalence of *A. actinomycetemcomitans* in Denmark may be lower than reported here.

In accordance with previous studies, we found an association between being positive for *A. actinomycetemcomitans* and having poorer periodontal health outcomes (Table 2 and Table 3) [2,3,4]. The association was primarily found in the group that was positive for *A. actinomycetemcomitans* in subgingival plaque and/or stimulated saliva, and in the group with no *A. actinomycetemcomitans* (Table 2 and Table 3). These findings may indicate that a pronounced occurrence of *A. actinomycetemcomitans* in an individual (with detection of *A. actinomycetemcomitans* in both subgingival plaque and stimulated saliva) is associated with poorer periodontal health outcomes. However, due to the study being cross-sectional, we cannot conclude whether *A. actinomycetemcomitans* is part of the pathogenesis leading to poorer periodontal health outcomes or if the presence of *A. actinomycetemcomitans* is a consequence of disease.

The study population consisted mainly of very periodontally healthy individuals, and the proportion of individuals with a non-Danish ethnic background was higher than in the general Danish population. However, the diversity of ethnicity in the present study population represented the adolescent population of the municipality of Aarhus (Table 1).

The bacterial load was not quantified, in contrast to what has been done by other researchers [9,18,36,37]. Still, we used valid sampling methods (pooled subgingival plaque samples and stimulated saliva samples) and sensitive detection methods (real-time PCR), which have also been used in other studies [38,39,40,41]. Therefore, we are confident in reporting a prevalence of 13% *A. actinomycetemcomitans*-carriers in the present study. Finally, due to the study design and the participants, the calibration process of the examiner was performed on healthy individuals, and it is well known that it is easier to reproduce a measurement of “zero”.

Despite the limitations addressed above, the findings presented here justify further research on *A. actinomycetemcomitans* and its implication in a Danish adolescent population. The JP2 genotype of *A. actinomycetemcomitans* was not detected in the present study population. However, studies on the prevalence of *A. actinomycetemcomitans* are still of importance due to the association of this microorganism with periodontitis and non-oral diseases [27]. The presence of *A. actinomycetemcomitans* among primarily healthy Danish adolescents is indisputable, and the results of the present study suggest a positive association between being positive for *A. actinomycetemcomitans* in both subgingival plaque and stimulated saliva and having poorer periodontal health outcomes in the Danish population.

## 4. Materials and Methods

### 4.1. Study Population

In the year 2018, 3165 14- to 15-year-old individuals were affiliated with the dental clinics of the municipality of Aarhus, Denmark. With the aim of examining a 25% sample of the 14- to 15-year-old individuals affiliated with the municipality of Aarhus, 1145 (1/3 of the population of 14- to 15-year-olds) were randomly selected for enrolment in the present cross-sectional study. The criteria for participation were Danish citizenship, being born in the year 2003, and having had no prior dental examination during the 2018 calendar year. Out of the 1145 selected individuals, 220 (19.2%) adolescents had unintentionally already had their routinely and mandatory clinical examinations carried out by the clinical staff of the municipality before enrolment. Therefore, 917 (80.1%) of the original sample were invited to participate in the present study by email as a part of their regular invitation to the mandatory examination at the age of 15. Of the 648 who attended the clinics for their mandatory examination, 525 (approximately 60% of the invited participants) individuals agreed to participate in the clinical study. The participants were equally distributed according to all parts of the municipality of Aarhus, comprising six districts. The demographic data concerning the ethnic background of the participants according to the districts were provided by the municipality of Aarhus [26].

The study was approved by the ethics committee of the central region of Denmark (regionmidtjylland) (1-10-72-385-17) (date of approval: 10-01-2018). Written informed consent was obtained from all participants.

### 4.2. Clinical Examination

The clinical examinations were carried out at the dental clinics in the municipality of Aarhus, with which the participants were affiliated. Six different districts and sixteen different clinics were visited during the year 2018 while carrying out the data collection process. One examiner (ABJ) carried out the clinical examinations, which included periodontal measurements of all fully erupted permanent teeth present in the oral cavity. Furthermore, all participants were asked to answer a questionnaire concerning oral hygiene habits, daily intake of medicine, smoking habits, family history of periodontal disease, and consumption of antibiotics within the last three months. All periodontal recordings were carried out on six sites per tooth (mesio-facial, facial, disto-facial, mesio-oral, oral, and disto-oral) by the use of a Deppeler™ periodontal probe (Deppeler SA, A-One Business Center, La Pièce 6,CH-1180 Rolle, Switzerland) (HH12FMS, 3-6-9-12 mm, 0.48 Ø). PS and BOP were recorded as present or not present. PPD was recorded from the gingival margin to the bottom of the periodontal pocket. CAL was recorded from the cemento-enamel junction to the bottom of the clinical periodontal pocket. All recordings were measured to the nearest millimeter.

Eleven individuals agreed to be examined subsequently within 48 hours, with the purpose of performing intra-examiner calibration. All six sites on all permanent teeth present were measured on both occasions. The calibration process was undertaken under the same conditions as the clinical examination in the clinical study. The intra-examiner reproducibility was 99.9% for PPD and 99.4% for CAL, with a margin-off-error of ± 1 mm.

### 4.3. Periodontal Outcomes

The cut-off level for having poor oral health defined by the PS and BOP was a PS > 20% of the sites and BOP > 10% of sites, respectively [42]. Only interdental CAL was used to describe the periodontal health of the individuals, because the main part of the facial and buccal clinical attachment loss was without additional PPD > 3 mm. Only one individual presented with facial and buccal PPD ≥ 4 mm in addition to CAL, defining a periodontitis lesion [43]. The cut-off for a deep PPD was set at 4 mm or more [42,44]. According to the new consensus for the diagnosis of periodontal disease, it is important to recognize CAL at one mm in the diagnosis of periodontitis, so that milder cases are not overlooked [43]. However, in epidemiological studies a cut-off above one mm is needed because of the margin of error of ±1 mm in measuring CAL. Therefore, we chose a cut-off of 2 mm in the present study in order not to miss early cases of periodontal disease in this young and otherwise healthy adolescent Danish population.

### 4.4. Biological Samples

#### 4.4.1. Stimulated Saliva Samples

Stimulated saliva samples were collected as described by Ennibi and coworkers [39]. Saliva from each participant was collected before the clinical examination. The participants were asked to chew a Vivoclar Vivadent Clinical paraffin tablet (Ivoclar Vivadent AG, Schaan, Liechenstein) for one minute. Subsequently, saliva was collected in a plastic beverage cup. One mL of the saliva was transferred into a sterile plastic tube with a screw cap, containing 1 mL of saliva preservation buffer (Norgen Biotek Corporation, Schmon Parkway Thorol, ON, Canada). Samples were transported to the Department of Clinical Microbiology, Aarhus University Hospital, Skejby, Denmark, and stored at room temperature. DNA from the saliva samples was purified using a Magna Pure 96 DNA and Viral NA (Large Kit) (Roche Diagnostics, GmbH, Mannheim, Germany) on Magna Pure 96 (Roche, Germany). The bacterial DNA was stored at −20 °C until being analyzed by real-time polymerase chain reaction (PCR) for the detection of *A. actinomycetemcomitans*.

#### 4.4.2. Subgingival Plaque Samples

Subgingival plaque samples were collected as one pooled subgingival sample from the mesio-facial sites of the permanent first molars before the clinical examination was carried out. Visual supragingival plaque was gently removed, and the teeth were kept dry using cotton rolls and dental suction, before a sterile paper point (Orbis Absorbent Paper Points, sterile, .04/40) (Obiliss.s, Brussels, Belgium) was placed in the periodontal pockets for 10–15 seconds. Paper points with biological samples were placed in tubes containing 1 mL of 0.9% saline. Samples were stored at 5 °C before being transported to the Department of Dentistry and Oral Health, Aarhus University, Aarhus, Denmark, where the samples were processed and bacterial DNA was purified. The purified bacterial DNA was stored at −20 °C until being analyzed by real-time PCR for the detection of *A. actinomycetemcomitans*.

#### 4.4.3. Real-Time Polymerase Chain Reaction

The detection of *A. actinomycetemcomitans* was performed as described by Marín et al. with a few modifications [45]. Briefly, bacterial DNA was analyzed using probe (5´-6FAM-AGA ACT CAG AGA TGG GTT TGT GCC TTA GGG-BBO-3´) and primers (forward: 5′-GAA CCT TAC CTA CTC TTG ACA TCC GAA-3′ and reverse: 5′-TGC AGC ACC TGT CTC AAA GC-3′) in a final volume of 15 µL PCR reaction mix containing 200 nM probe, 200 nM of each primer, 7.5 µL TaqMan Fast Advanced mastermix (Life Technologies by Thermoficher Scientific, Waltham, MA, USA), and 5 µL bacterial DNA. The PCR reaction mix was placed in a clear LightCycler® 480 Multiwell Plate 96 (Roche Diagnostics GmbH, Mannheim, Germany), and the plates were loaded into a LightCycler® 480 II (Roche Diagnostics International Ltd., Rotkreuz, Switzerland). Amplification reactions consisted of 10 min at 95 °C, followed by 40 cycles of 15 s at 95 °C and 1 min at 60 °C. Amplification, detection, and data analysis was performed with the LightCycler 480II system (Roche, Mannheim, Germany). Fluorescence was measured during the extension/annealing step of each cycle. D7SS served as the positive control, and H_2_O served as the negative control.

The bacterial samples positive for *A. actinomycetemcomitans* were additionally analyzed for the presence of the 530-bp deletion in the promoter region of the operon coding for the production of leukotoxin, characterizing the JP2 genotype of *A. actinomycetemcomitans*. Primers used were from Orrù et al. 2006 [41]. The real-time PCR was performed using a LightCycler instrument® 480 II (Roche Diagnostics International Ltd., Rotkreuz, Switzerland) and AmpliTaq Gold™ mastermix (appliedbiosystems by Thermo Fischer Scientific, Vilnius, Lithuania). The final volume of 20 µL contained 10 µL AmpliTaq Gold mastermix, 200 nM of each primer OG155 (5´- CATTCTCGGCGAAAAAACTA -3´) and OG156 (5´- CCCATAACCAAGCCACATAC -3´), 250 mM Syto 82 (Invitrogen by ThemoFisher Scientific), and 2 µL of bacterial DNA.

The PCR program was as follows. Denaturation was done at 95 °C for 10 min, followed by 50 cycles of 1 sec at 95 °C, 10 sec at 49 °C, 40 sec at 72 °C, and 3 sec at 74 °C. A melting curve was performed for 1 sec at 95 °C, 1 sec at 45 °C, followed by rise in temperature of 0.06 °C/sec up to 95 °C. Fluorescence was detected at the end of the 74 °C segment in the PCR step (single mode), and at the 45–95 °C segment in the melting step (continuous mode) in the VIC (Hex, Joe) channel.

Melting curve peaks were observed at 74.3–74.4 for JP2 genotypes and 77.4–76.7 for non-JP2 genotypes. Strain JP2 (HK921) served as the positive control and H_2_O served as the negative control.

### 4.5. Statistical Analysis

The statistical analysis was performed using STATA 15 (StataCorp. 2017. *Stata Statistical Software: Release 15*. College Station, TX: StataCorp LLC.) and SciPy [46], which is an open source scientific tool for Python® (Beaverton, OR, USA). The chi squared test was used to compare the periodontal health outcomes between the groups determined according to carrier status of *A. actinomycetemcomitans*. A proportion test was used to analyze the statistical difference between the periodontal health outcomes of the group of individuals positive for *A. actinomycetemcomitans* in subgingival plaque only and the group of individuals positive for *A. actinomycetemcomitans* in stimulated saliva only. Intra-examiner reproducibility was calculated as a percentage of total agreement and with a margin of error of ± 1 mm between two measurements of PPD and CAL.

## Figures and Tables

**Table 1:** Demographic characteristics of the six districts of the municipality of Aarhus.

No. of District of Municipality of Aarhus, Denmark	No. of Participants (%)	District Size According to the Total no. of Individuals Born in Year 2003 in the Municipality (%)	Proportion of Individuals with a Danish Ethnic Background (%)
1	93 (17.7)	16.9	93.9
2	72 (13.7)	13.9	76.9
3	95 (18.1)	18.0	59.4
4	102 (19.4)	17.7	90.4
5	96 (18.3)	18.5	75.0
6	67 (12.7)	14.9	84.0

**Table 2:** Periodontal outcomes of the group of individuals positive for *A. actinomycetemcomitans* (*Aa*) and the group of individuals without *A. actinomycetemcomitans*.

Periodontal Outcomes	Individuals Positive for *Aa* (*n* = 70)	Individuals with no *Aa* (*n* = 455)	Total (*n* = 525)
**No. of individuals with PS ^1^ > 20% (%)**	54 (77.1)	327 (71.9)	381 (72.6)
**No. of individuals with BOP ^2^ > 10% (%)**	66 (94.3)	431 (94.6)	497 (94.7)
**No. of individuals with PPD ^3^ ≥ 4 mm (%)**	26 (37.1)*	83 (18.2) *	109 (20.0)
**No. of individuals with interdental CAL ^4^ ≥ 2 mm (%)**	4 (5.7)*	5 (1.1) *	9 (1.7)

* Statistical significance between groups (*p* < 0.05). ^1^ plaque scores, ^2^ bleeding on probing, ^3^ periodontal probing depth, ^4^ clinical attachment loss.

**Table 3:** Periodontal outcomes according to carrier status of *A. actinomycetemcomitans* (*Aa*).

Periodontal Outcomes	Individuals Positive for *Aa* in Subgingival Plaque (*n* = 51)	Individuals Positive for *Aa* in Stimulated Saliva (*n* = 54)	Individuals Positive for *Aa* in Subgingival Plaque only (*n* = 16)	Individuals Positive for *Aa* in Saliva only (*n* = 19)	Individuals Positive for *Aa* in both Saliva and Subgingival Plaque (*n* = 35)	Individuals with no *Aa* (*n* = 455)
**No. of individuals with PS ^1^ > 20% (%)**	44 (86.3) *	42 (77.8)	12 (75.0)	10 (52.6)	32 (91.4) *	327 (71.9)
**No. of individuals with BOP ^2^ > 10% (%)**	50 (98.0)	51 (94.4)	15 (93.6)	16 (84.2)	35 (100)	431 (94.6)
**No. of individuals with PPD ^3^ ≥ 4 mm (%)**	20 (39.2) *	22 (40.7)*	4 (25.0)	6 (31.6)	16 (45.7) *	83 (18.2) *
**No. of individuals with interdental CAL ^4^ ≥ 2 mm (%)**	4 (7.8) *	3 (5.6)*	1 (6.3)	0 (0.0)	3 (8.6) *	5 (1.1) *

Statistical significance between groups (*p* < 0.05). ^1^ plaque scores, ^2^ bleeding on probing, ^3^ periodontal probing depth, ^4^ clinical attachment loss.
